# Muscle and tendon morphology of a world strongman and deadlift champion

**DOI:** 10.1152/japplphysiol.00342.2024

**Published:** 2024-08-15

**Authors:** Thomas G. Balshaw, Garry J. Massey, Robert Miller, Emmet J. McDermott, Thomas M. Maden-Wilkinson, Jonathan P. Folland

**Affiliations:** ^1^School of Sport, Exercise, and Health Sciences, Loughborough University, Loughborough, United Kingdom; ^2^College of Life and Environmental Sciences, University of Exeter, Exeter, United Kingdom; ^3^UK Athletics, Loughborough University, Loughborough, United Kingdom; ^4^Department of Sport Science, Aspire Academy, Doha, Qatar; ^5^Department of Physical Education and Sport Sciences, University of Limerick, Limerick, Ireland; ^6^Academy of Sport and Physical Activity, Faculty of Health and Wellbeing, Sheffield Hallam University, Sheffield, United Kingdom

**Keywords:** isometric force, magnetic resonance imaging, power, strength

## Abstract

This study compared the muscle and tendon morphology of an extraordinarily strong individual, a World’s Strongest Man and deadlift champion (WSM), with that of various other athletic, trained, and untrained populations. The WSM completed the following: *1*) 3.0-T MRI scans, to determine the volume of 22 individual lower limb muscles, 5 functional muscle groups, patellar tendon (PT) cross-sectional area (CSA), and PT moment arm; and *2*) countermovement jumps (CMJ) and isometric midthigh pull (IMTP) contractions. The WSM was compared with previously assessed groups from our laboratory (muscle and tendon) and the wider research literature (CMJ and IMTP). The WSM’s CMJ peak power (9,866 W) and gross (9,171 N) and net (7,480 N) IMTP peak forces were higher than any previously published values. The WSM’s overall measured leg muscle volume was approximately twice that of untrained controls (+96%) but with pronounced anatomical variability in the extent of muscular development. The plantar flexor group (+120%) and the guy rope muscles (sartorius, gracilis, and semitendinosus: +140% to +202%), which stabilize the pelvis and femur, demonstrated the largest differences relative to that of untrained controls. The WSM’s pronounced quadriceps size (greater than or equal to twofold vs. untrained) was accompanied by modest PT moment arm differences and, notably, was not matched by an equivalent difference in PT CSA (+30%). These results provide novel insight into the musculotendinous characteristics of an extraordinarily strong individual, which may be toward the upper limit of human variation, such that the WSM’s very pronounced lower limb muscularity also exhibited distinct anatomical variability and with muscle size largely uncoupled from tendon size.

**NEW & NOTEWORTHY** Lower-body muscle size of an extraordinarily strong individual, a World's Strongest Man and deadlift champion (WSM), was approximately twice that of controls but was underpinned by pronounced anatomical variability in the extent of muscular development (+23–202%): the plantar flexor group and guy rope muscles demonstrating the largest differences. The WSM’s quadriceps size (more than or equal to twice that of controls) contrasted with modest differences in patella tendon moment arm (+18%) and was uncoupled from patellar tendon size (+30%).

## Graphical abstract

**Figure:**
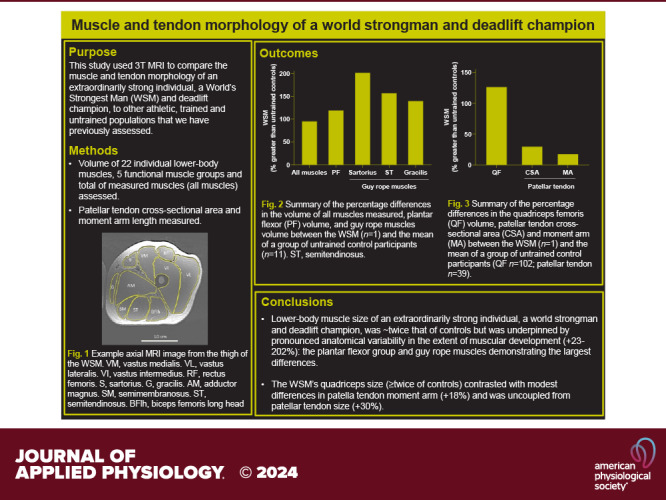

## INTRODUCTION

Feats of strength have fascinated man since the early stages of human civilization, as shown by the archeological evidence of inscribed heavy stones at Olympia and Thera in Greece, dated to the 6th century BC, detailing the way they were lifted by Bybon and Eumastus, respectively (1). Over the centuries, many types of strength competitions have existed; some of which have been codified and endured within modern sporting competitions (e.g., weightlifting, powerlifting, and shot put). In addition, professional strongman competitions, such as the annually contested “World’s Strongest Man” event, generate extensive global interest (2). Moreover, scientific understanding of muscular strength is important because of its role in athletic performance (3), injury prevention (4), and healthy aging (5). However, our knowledge of extreme human strength is limited.

To date, there is little scientific information on the characteristics of extremely strong humans in terms of laboratory-based tests of strength and power, particularly the size and distribution of their muscle mass, as well as tendon size and joint mechanics (moment arm). Kraemer et al. (6) examined the body composition of elite strongman competitors using dual-energy X-ray absorptiometry scanning and found that they had a body mass (153 ± 19 kg) and lean mass (118 ± 12 kg) approximately twice that of an average untrained healthy young man. Whole body skeletal muscle mass of athletes from strength- and power-based sports has also been estimated using ultrasound measurements at a limited number of anatomical locations (7, 8). However, neither ultrasound-derived predictions of skeletal muscle mass nor dual-energy X-ray absorptiometry provides detailed information on the size of specific individual muscles. Given the known importance of muscle size as a determinant of muscular strength (9–11), pronounced muscle size seems likely to be critical to extreme human strength; however, the specific muscle size of extremely strong individuals remains unknown. Similarly, a large moment arm (e.g., of the patella tendon at the knee joint) could contribute to the expression of high muscular strength (10, 12), and a large tendon may mitigate the mechanical stress it experiences with very high muscular loads, and therefore, these characteristics may also be expected in individuals selected for exceptional strength.

In this paper, we present the findings from a unique opportunity to examine the laboratory function, muscle size, and distribution of muscle mass, as well as patellar tendon size and moment arm, of a World's Strongest Man and deadlift champion (WSM) in comparison with existing data on untrained individuals, power athletes (100-m-track sprinters), and long-term resistance-trained populations that we have assessed previously (10, 11, 13–15).

## MATERIALS AND METHODS

### Participant

The WSM’s achievements included one World’s Strongest Man title (14 mo prior to measurement), five Britain’s Strongest Man titles (the most recent 6 mo prior to measurement), twice being World Deadlift Champion and Deadlift World Record holder (500 kg; at the time of measurement), and second place at Europe’s Strongest Man. Prior to agreeing to participate, the purpose of the research study and the testing procedures were explained to the participant along with the risks and benefits of taking part. The participant gave his written informed consent to participate in the study that was approved by the Loughborough University Ethical Advisory Committee (Ethics Number R18-P090). Included in the written consent was a statement providing permission for publication of the collected data and the likelihood that their identity may be evident based on their achievements and characteristics, despite anonymization.

### Training History

The WSM had been continuously involved in systematic, regular upper- and lower-body resistance training for 15 yr at the time of testing. In the 12 mo prior to testing, the participant’s resistance training consisted of the following typical exercises: lower body: squats, deadlifts, leg press, and knee extension; and upper body: bench press, shoulder press, dumbbell/barbell rows, and lat pull-down. The proportion of the participant’s training within the following repetition ranges over the last 12 mo was as follows: near maximum loads [1–5 repetition maximum (RM)]: 10%; heavy loads (6–14 RM): 80%; and moderate loads (≥15 RM): 10%. The participant reported only occasional (<1×/week) use of advanced resistance training practices (i.e., complex training and accommodating resistance method) but frequently (>3×/week) executed training repetitions with the intention to move the load as fast as possible. The WSM’s nutritional supplement consumption included protein, branched-chain amino acids, and electrolytes.

### Overview

The WSM reported for a single test session that involved the following assessments (listed in order): axial T1 weighted 3.0-T MRI scans from T12 to the lateral malleolus [to assess muscle size throughout the lower body (left and right sides)], axial and sagittal T1-weighted MRI scans of both knees [to assess patellar tendon cross-sectional area (CSA) and patellar tendon moment arm], maximum countermovement jumps (CMJ), and maximum isometric midthigh pulls (IMTPs). The muscle size, patellar tendon CSA, and patellar tendon moment arm of the WSM were compared with various populations measured within our laboratory, as indicated in Table 1, alongside participant descriptives (10, 11, 13–15). In addition, the IMTP and CMJ measures were compared with existing published literature (included studies are summarized in Supplemental Materials 1 and 2, alongside participant descriptives).

**Table 1.** Descriptive characteristics of a World's Strongest Man and deadlift champion and populations featured within this study for the purposes of providing comparative muscle and tendon morphology data

	*n*	Age, yr	Height, m	Body Mass, kg	Source of Comparative Data
WSM	1	30.6	1.90	172.0	
Overall muscle morphology
Elite sprint runners	5	27.4 ± 4.1	1.83 ± 0.06	86.4 ± 6.7	Miller et al. (13)
Subelite sprint runners	26	22.0 ± 2.2	1.78 ± 0.06	75.4 ± 7.3	
Untrained controls	11	25.8 ± 2.6	1.80 ± 0.08	75.2 ± 5.6	
Quadriceps femoris muscle morphology
Long-term resistance-trained	16	22 ± 2	1.83 ± 0.06	91 ± 10	Maden-Wilkinson et al. (10)
Untrained controls	102	25 ± 3	1.78 ± 0.08	73 ± 10	Pooled sample from Miller et al. (13) (*n* = 11), Balshaw et al. (11) (*n* = 52), and pretest of Balshaw et al. (14) (*n* = 39)
Hamstrings muscle morphology
Long-term resistance-trained	16	22 ± 2	1.83 ± 0.06	91 ± 10	Unpublished observations from the sample in Maden-Wilkinson et al. (10)
Untrained controls	50	26 ± 4	1.79 ± 0.08	75 ± 11	Pooled sample from Miller et al. (13) (*n* = 11) and pretest of Balshaw et al. (14) (*n* = 39)
Patellar tendon CSA and moment arm
Long-term resistance-trained	16	22 ± 2	1.83 ± 0.06	90 ± 10	Massey et al. (15)
Untrained controls	39	25 ± 2	1.76 ± 0.06	72 ± 9	

Values for comparative populations are means ± SD. CSA, cross-sectional area.

### MRI Measurement of Muscle Tendon Unit Morphology and Moment Arm

The participant reported for their MRI scan [3.0-T Discovery MR750W (70-cm-wide bore), GE Medical] having not completed any strenuous physical activity in ≥24 h and had received prior instruction to arrive in a relaxed state having eaten and drunk normally. The participant sat quietly for 15 min prior to their scan. The participant lay supine for the MRI scan of the lower-body musculature from T12 to the lateral malleolus. A body coil (GE Medical) allowed axial T1-weighted images (time of repetition/time to echo 600/8.144 ms, image matrix 512 × 512, field of view 500 × 500 mm, pixel size 0.9766 × 0.9766 mm, slice thickness 5 mm, and interslice gap 5 mm) to be acquired in five overlapping blocks. Images of both sides of the body were acquired within a single scan for blocks 1 (T12 to pelvis), 4 (knee joint space to midshank), and 5 (midshank to lateral malleolus). However, due to the size of the participant’s thighs, it was necessary to scan each thigh individually for blocks 2 (pelvis to midthigh) and 3 (midthigh to knee joint space); this involved the radiographer repositioning the field of view between scanning the first and the second thigh but not physically moving the coil or the participant. Oil-filled capsules were secured to the surface of the participant’s skin with Transpore tape at intervals along the length of the lower body prior to the scan and in an offline analysis used to verify the alignment of the blocks (Horos software, Version 3.36, https://horosproject.org/).

**Figure 1.**
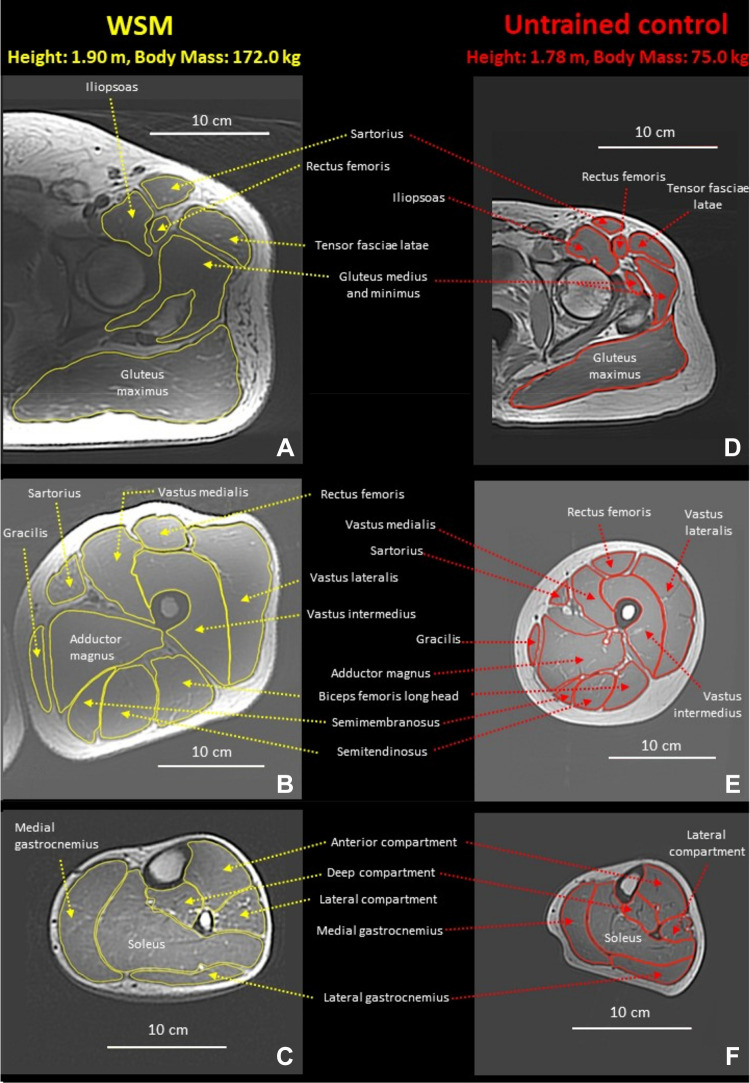
Example axial MRI images from the World's Strongest Man and deadlift champion (WSM; *A*–*C*) and an untrained control participant (*D*–*F*) from the hip (*A* and *D*), thigh (*B* and *E*), and lower leg (*C* and *F*). Image location relative to femur and shank length was matched between the WSM and the untrained control as follows: hip image is at approximately midfemoral head, thigh image is at ∼52% of femur length (0% is distal end of femur, 100% is greater trochanter), and lower leg image is at ∼70% of shank length (0% is lateral malleolus, 100% is proximal end of tibia). The untrained control participant displayed was from the work by Miller et al. (13) and had a total measured muscle volume of all measured muscles that was 5.1% smaller than the mean of the untrained group within that study.

The offline analysis was of the following muscles/compartments (Fig. 1): iliopsoas (psoas major and iliacus combined); sartorius; tensor fasciae latae; adductor magnus; gracilis; gluteus maximus; gluteus medius and minimus (combined, due to difficulty separating the two muscles); rectus femoris (RF); vastus lateralis (VL), medialis (VM), and intermedius (VI); semimembranosus (SM); semitendinosus (ST); biceps femoris long (BFlh) and short heads (BFsh); popliteus; lateral and medial gastrocnemius; soleus; and the anterior, lateral, and deep posterior compartments of the shank. The anterior shank compartment consisted of the tibialis anterior, extensor digitorum longus, and extensor hallucis longus. The lateral shank compartment included the peroneus longus and brevis. The deep posterior compartment consisted of plantaris, tibialis posterior, flexor digitorum longus, and flexor hallucis longus. All muscles were manually segmented in every other image (i.e., every 20 mm) starting from the most proximal image in which the muscle appeared, except the tensor fasciae latae, gluteus medius and minimus (combined), and popliteus, which were manually segmented in every slice (i.e., every 10 mm) due to their short length. The volume of each individual muscle (*V*_m_) was calculated using previously outlined methods (16) as follows:

$$V_{\text{m}}=\sum_{i=1}^{n-1}\frac{h}{2}(A_{mi}+A_{m\mathrm{i+}1})$$

where *A*_m_ represents the muscle CSA calculated from each image, *i* is the image number, *n* is the total number of images, and *h* is the distance between images. The volume of five functional muscle groups was calculated as the sum of the following muscles: hip extensors (gluteus maximus, adductor magnus, BFlh, SM, and ST), hip flexors (iliopsoas, RF, sartorius, and tensor fasciae latae), knee extensors (RF, VI, VM, and VL), knee flexors (gracilis, BFlh and BFsh, SM, ST, sartorius, popliteus, and medial and lateral gastrocnemius), and plantarflexors (medial and lateral gastrocnemius and soleus). The sum of all the measured lower-body muscles was also quantified as the volume of “all muscles.”

Once muscle MRI scanning had been completed, a flex coil (GE Medical) was used to acquire unilateral T1-weighted axial (time of repetition/time to echo 650/9.476 ms, image matrix 512 × 512, field of view 180 × 180 mm, pixel size 0.3516 × 0.3516 mm, slice thickness 2 mm, and interslice gap 0 mm) and sagittal images (time of repetition/time to echo 606/9.512 ms, image matrix 512 × 512, field of view 180 × 180 mm, pixel size 0.3516 × 0.3516 mm, slice thickness 2 mm, and interslice gap = 0 mm) from both knee joints. The axial images were obtained perpendicular to the line of the tendon from ∼2 cm superior to the apex of the patella to ∼2 cm inferior to the patellar tendon’s inferior insertion. Patellar tendon CSA was measured in each contiguous image along the length of the tendon (i.e., from the first image where the patella was no longer visible to the final image before the tibial insertion). The axial images of the patellar tendon were viewed in grayscale, sharpened, and the perimeter manually outlined. The average of all measured axial patellar tendon CSAs was calculated to produce a mean tendon CSA (mm^2^) for each leg. The moment arm length of the patellar tendon for each leg was estimated from sagittal plane images as the perpendicular distance from the patellar tendon to the midpoint of tibiofemoral contact (17).

### Countermovement Jump

Following an ∼10-min self-selected whole body loaded barbell-based warm-up and three submaximum warm-up CMJs performed with ∼50% of perceived maximum effort, the WSM performed three maximal effort CMJs, with 30 s of rest between jumps, on a portable Kistler force plate (Quattro Jump, Type 9290AD, Kistler, Switzerland), interfaced with a personal computer. Prior to all jumps, the participant was instructed to stand still on the force plate in an upright posture with their arms by their sides. Sampling was initiated when they provided an indication they were ready to begin, and after a 2-s pause to collect the force due to body mass and a 3-s countdown, the participant performed a CMJ for maximal height, with arm movement and the depth of countermovement self-selected by the participant. The Quattro jump device records vertical ground reaction force at a sampling frequency of 500 Hz and calculates jump height and peak power using integration/double integration of the force signal (Quattro jump software, type 2822A1-1, version 1.1.1.4) to derive velocity (multiplied by force to calculate power) and displacement (jump height).

### Isometric Midthigh Pull

IMTP contractions were performed within an isometric rig consisting of a base plate with stainless steel uprights (ESP Fitness, Loughborough, UK), which facilitated barbell adjustment to different heights. A bar height producing a knee joint angle of 145° (measured by a manual goniometer) was selected, and the participant was instructed to keep his torso upright while completing the IMTP efforts. Two calibrated 10-kN-capacity force platforms (model 9286B, Kistler Instruments, Ltd., London, UK), one underneath each foot, were placed on top of the isometric rig’s base plate, and vertical force signals from the eight individual load cells across the two force platforms were outputted (External Control Unit model 5233 A, Kistler Instruments, Ltd.) and sampled at 2,000 Hz using an external analog-to-digital converter (Micro 1401; CED, Cambridge, UK) and recorded with Spike 2 computer software (CED, Cambridge, UK).

Following a warm-up consisting of a series of incremental warm-up contractions of ∼5 s duration ranging from 50% to 90% of maximum perceived effort, two maximum IMTP efforts of 3–5 s duration were performed under the instruction to “pull as hard as possible.” Six minutes separated the maximum efforts, based on a self-selected recovery period. Wrist wraps were worn to remove the influence of grip strength from the assessment. Real-time overall feedback from the force platforms (the sum of the force signals from the load cells across the two platforms) was displayed in front of the participant during the IMTP efforts, and a horizontal marker was placed on the highest force obtained after the first maximum effort. In the offline analysis, the force signals were low pass filtered (10 Hz using a fourth-order zero-lag Butterworth filter) before summating the force output from the two platforms to derive overall force produced. The instantaneous highest force during maximum efforts was identified as the measure of gross IMTP peak force (i.e., including body weight). Force while the WSM was standing upright on the platform at rest (i.e., body weight) was also subtracted from the peak instantaneous force to calculate net IMTP peak force.

### Analysis and Comparative Data

Muscle volumes, patellar tendon CSA, and patellar tendon moment arm measurements assessed on both legs of the WSM were averaged to provide unilateral criterion values; this facilitated comparisons with various untrained, resistance-trained, and athletic groups previously investigated in published works from our laboratory (10, 11, 13–15; Table 1). IMTP and CMJ values were predominantly compared with existing research literature with the highest comparable male data [e.g., IMTP gross peak force: (18–25); IMTP net peak force: (26–31); CMJ performed with an arm swing on a force platform (32–38)]. Where the numerical values (means and SD) from previously published studies were not reported, they were extracted using online software (WebPlotDigitizer, version 4.6, https://automeris.io/WebPlotDigitizer). For IMTP peak force in cases where it was not clearly stated that body weight was subtracted from gross IMTP peak force, measures were assumed to be gross IMTP peak force. Muscle and tendon morphology figures display means ± SD as well as individual participant data for comparative populations, as these values are from published research from our laboratory. IMTP peak force and CMJ outcome figures display only means ± SD values for comparative populations, as we relied on published values from the literature where individual participant values were not typically available.

## RESULTS

### Participant Descriptives and Anthropometrics

The WSM was 30.6 yr old and 1.90 m tall and his body mass was 172 kg upon reporting for the laboratory visit. The age, height, and body mass of participants from the comparative datasets featured in our previously published research are presented in Table 1. Age, height, and body mass for comparative populations drawn from the existing literature can be found in Supplemental Materials 1 (gross IMTP peak force and net IMTP peak force) and 2 (CMJ peak power and height).

### Isometric Midthigh Pull and Countermovement Jump

**Figure 2.**
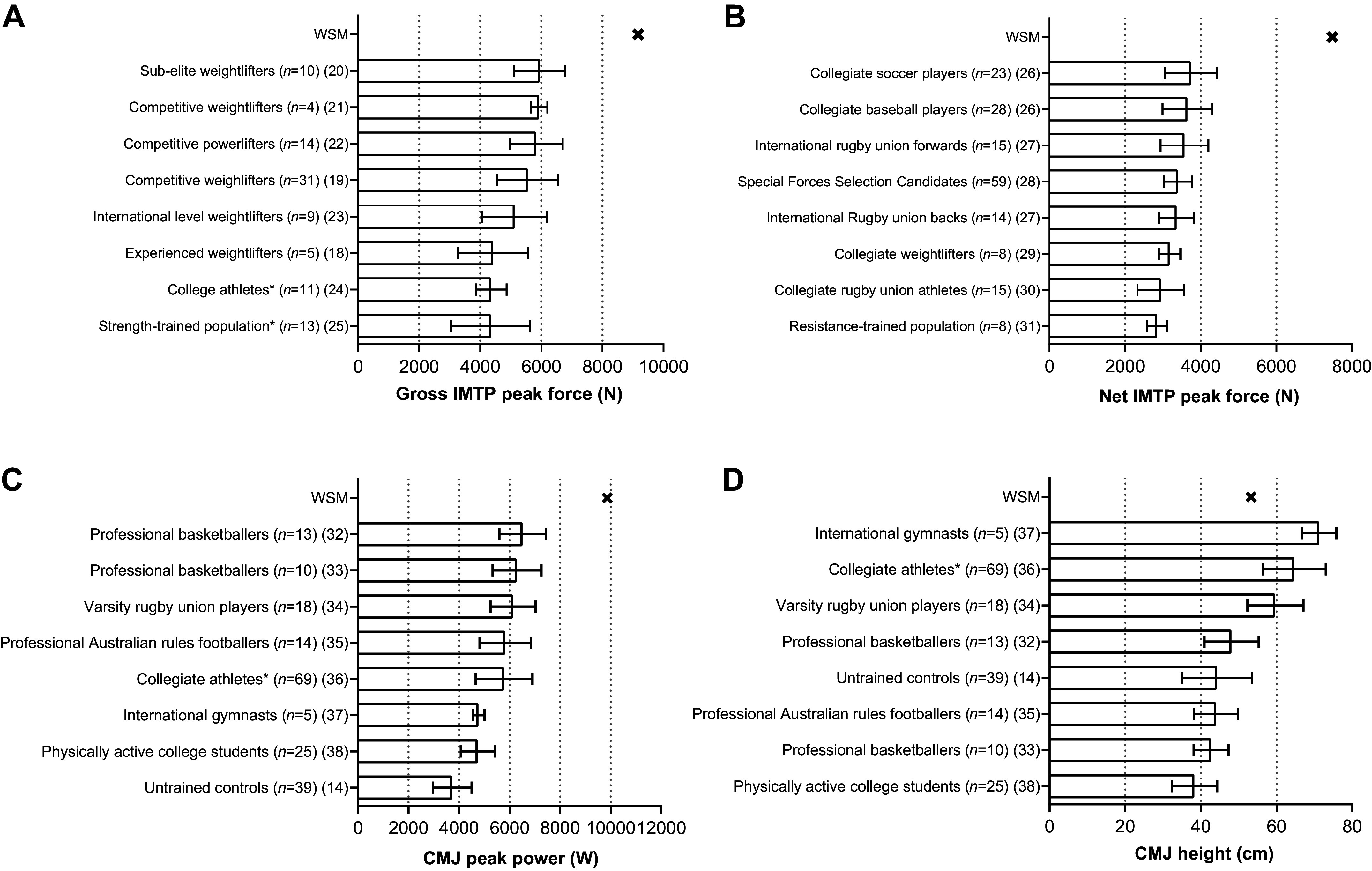
Gross (including body weight) isometric midthigh pull (IMTP) peak force (*A*), net (above body weight) IMTP peak force (*B*), countermovement jump (CMJ) peak power (*C*), and CMJ height (*D*) of a World's Strongest Man and deadlift champion (WSM) displayed against comparative data from the existing research literature. CMJ was performed with an arm swing by WSM and within all comparative data included in the figure. *Athletes from different sports or disciplines featured within the sample. Descriptive information (age, height, and body mass) of the groups included as comparative data can be found in Supplemental Materials 1 (IMTP) and 2 (CMJ).

Gross (including body weight) and net (above body weight) IMTP peak forces of the WSM were 9,171 N and 7,480 N, respectively. The WSM’s gross IMTP peak force was 54% greater than the highest comparable group mean we located (subelite weightlifters: 5,942 ± 844 N (20); Fig. 2*A*). The WSM’s net IMTP peak force was 100% greater than the highest comparable group mean value in the literature (collegiate soccer athletes: 3,740 ± 692 N (26); Fig. 2*B*).

The WSM’s CMJ peak power and jump height were 9,866 W and 53.3 cm, respectively. The peak CMJ power of the WSM was >2.5-fold (164%) that of the mean of an untrained control group previously measured in our laboratory (3,735 ± 760 W; unpublished) and 51% greater than the highest comparable group mean value we located in the literature (professional basketball players: 6,518 ± 923 W (32); Fig. 2*C*). Not surprisingly, given the WSM’s high body mass, his jump height was less exceptional, while still being 20% greater than that of a group of untrained control participants previously measured in our laboratory (44.3 ± 9.2 cm; unpublished). However, his jump height was 25% lower than the highest group mean CMJ height we are aware of in the published literature (elite international gymnasts: 71.3 ± 4.5 cm (37); Fig. 2*D*).

### Leg Muscle Volumes

**Figure 3.**
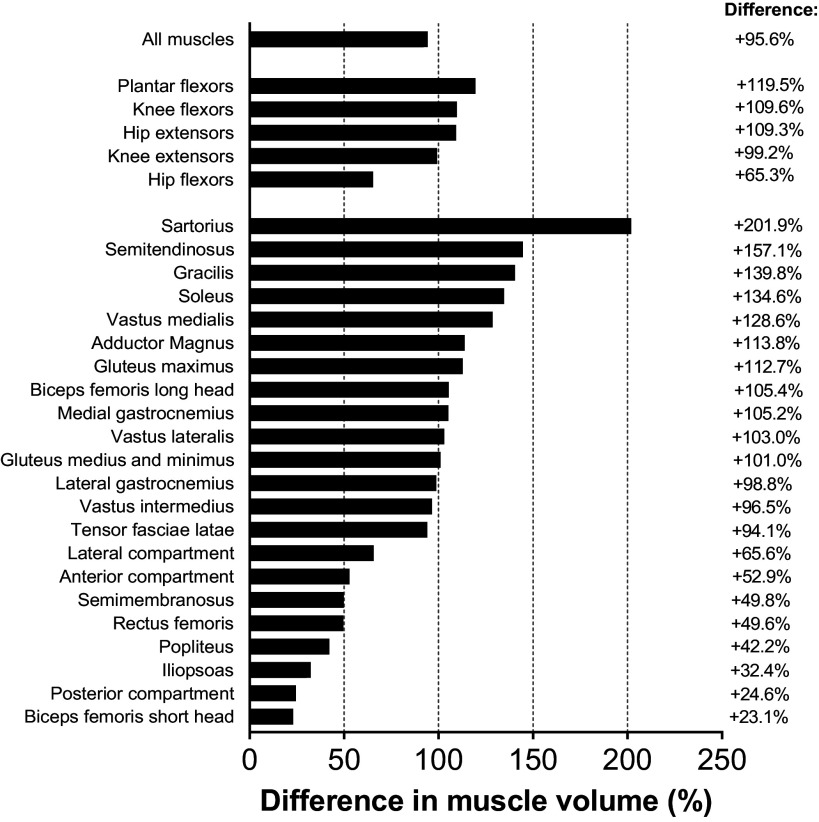
Percentage differences in muscle volumes of all muscles, 5 functional muscle groups, and 23 individual muscles/compartments between the World's Strongest Man and deadlift champion (WSM; *n* = 1) and untrained control participants (*n* = 11) from the work by Miller et al. (13). A positive value indicates greater muscle volume of WSM relative to the group mean of the untrained controls. The functional muscle groups and individual muscles are ordered according to the magnitude of the percentage differences for absolute muscle volume.

The total unilateral muscle volume of the 22 measured muscles/compartments of WSM (14,922 cm^3^) was nearly twice that of a relatively modest (*n* = 11) sample of untrained controls (7,628 ± 1,548 cm^3^; +96%; Fig. 3), while being 63% greater than subelite (9,164 ± 1,207 cm^3^) and +32% greater than elite 100-m sprinters (11,323 ± 1,328 cm^3^; Table 2). The muscle group differences were largest for the plantar flexors (+120% vs. untrained; +100% vs. subelite sprinters; +70% vs. elite sprinters) and smallest for the hip flexors (+65% vs. untrained; +30% vs. subelite sprinters; +5% vs. elite sprinters). The WSM had the highest values of any individual we have observed for four out of five muscle groups, but not the hip flexors, which were inferior to three of the elite 100-m sprinters (*n* = 5).

**Table 2.** Muscle volume of all muscles, 5 functional muscle groups, and 22 individual muscles/compartments of a World's Strongest Man and deadlift champion and comparative elite sprinters, subelite sprinters, and untrained control participants

Muscle Group/Muscle or Compartment	Muscle Volume, cm^3^			
	WSM	Elite Sprinters (*n* = 5)	Subelite Sprinters (*n* = 26)	Untrained (*n* = 11)
All muscles	14,922	11,323 ± 1,328	9,164 ± 1,207	7,628 ± 1,548
Hip flexors	1,704	1,620 ± 200	1,314 ± 216	1,031 ± 151
Hip extensors	4,724	4,002 ± 489	3,029 ± 422	2,257 ± 220
Knee flexors	3,060	2,304 ± 178	1,859 ± 301	1,460 ± 196
Knee extensors	4,386	3,218 ± 400	2,636 ± 401	2,202 ± 315
Plantar flexors	1,888	1,112 ± 181	943 ± 156	860 ± 172
				
Iliopsoas	681	702 ± 97	618 ± 101	514 ± 75
Sartorius	429	306 ± 46	209 ± 50	142 ± 25
Tensor fasciae latae	142	135 ± 41	86 ± 25	73 ± 24
Adductor magnus	1,334	1,056 ± 83	828 ± 128	624 ± 81
Gracilis	235	180 ± 37	142 ± 37	98 ± 23
Gluteus maximus	1,980	1,797 ± 376	1,257 ± 197	931 ± 108
Gluteus medius and minimus	1,172	626 ± 129	575 ± 97	583 ± 76
Rectus femoris	453	476 ± 45	401 ± 78	303 ± 55
Vastus lateralis	1,508	1,132 ± 180	925 ± 156	743 ± 98
Vastus intermedius	1,336	962 ± 145	789 ± 140	680 ± 115
Vastus medialis	1,088	649 ± 97	521 ± 79	476 ± 111
Semimembranosus	392	359 ± 60	327 ± 59	262 ± 18
Semitendinosus	563	449 ± 70	350 ± 79	219 ± 39
Biceps femoris long head	454	340 ± 31	267 ± 47	221 ± 42
Biceps femoris short head	135	167 ± 26	131 ± 34	110 ± 28
Popliteus	27	23 ± 5	17 ± 5	19 ± 6
Lateral gastrocnemius	310	202 ± 34	170 ± 37	156 ± 41
Medial gastrocnemius	515	300 ± 38	262 ± 58	251 ± 52
Soleus	1,063	610 ± 137	510 ± 76	453 ± 95
Anterior compartment	445	302 ± 59	273 ± 47	291 ± 47
Lateral compartment	253	147 ± 32	161 ± 42	153 ± 35
Posterior compartment	406	401 ± 76	345 ± 71	326 ± 93

Individual measurements are the average of both sides/legs (i.e., unilateral). All muscles are the sum of muscle volumes from all the individual muscles/compartments listed. Muscle volume data are presented as group means ± SD, except for the WSM (*n* = 1). Untrained control participants from Miller et al. (13).

Compared with untrained control participants (*n* = 11), all 22 of the WSM’s individual muscles/compartments were larger than untrained controls (Table 2 and Fig. 3). However, the differences in muscle volume were extremely variable, with the biggest differences being for the “guy ropes,” which were 2.5–3.0 times that of untrained controls (+140% gracilis; +157% ST; +202% sartorius), compared with more modest differences such as 23% (BFsh) and 32% (iliopsoas) greater.

### Quadriceps Femoris and Hamstring Size

**Figure 4.**
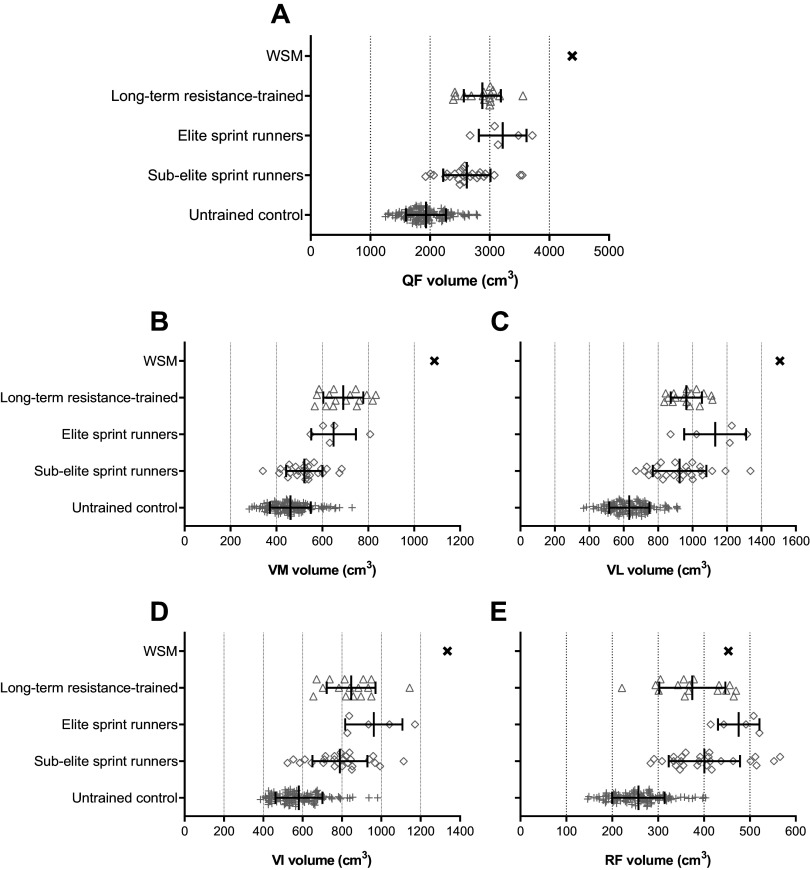
Quadriceps femoris (QF; *A*), vastus medialis (VM; *B*), vastus lateralis (VL; *C*), vastus intermedius (VI; *D*), and rectus femoris (RF; *E*) muscle volume of a World's Strongest Man and deadlift champion (WSM) compared with long-term resistance-trained (*n* = 16, from the work by Maden-Wilkinson et al. (10)], elite sprint runners [*n* = 5, from the work by Miller et al. (13)], subelite sprint runners [*n* = 26, from the work by Miller et al. (13)], and untrained control populations [*n* = 102, pooled population from the works by Miller et al. (13) (*n* = 11), Balshaw et al. (11) (*n* = 52), and Balshaw et al. (14) (pretest data *n* = 39)].

Overall quadriceps femoris volume of the WSM (4,386 cm^3^) was 127% greater than a large, pooled population of untrained controls (1,932 ± 336; *n* = 102), 66% greater than subelite sprinters (2,636 ± 401 cm^3^), 53% greater than long-term resistance-trained individuals (2,876 ± 311 cm^3^), and 36% greater than elite sprinters (3,218 ± 400 cm^3^; Fig. 4*A*). Moreover, the WSM’s quadriceps femoris was 18% larger than the most muscular individual we have previously assessed (elite sprinter: 3,716 cm^3^). The volumes of the individual vasti muscles of the WSM (VL: 1,508 cm^3^; VI: 1,336 cm^3^; VM: 1,088 cm^3^) were 130–138% larger than untrained controls (VL: 633 ± 117 cm^3^; VI: 581 ± 120 cm^3^; VM: 461 ± 89 cm^3^) and also greater than any trained/athletic individual we have previously assessed (Fig. 4, *B*–*D*). However, the WSM’s RF (453 cm^3^) was not quite so large, being 76% greater than untrained controls (257 ± 57 cm^3^) but smaller than the average elite sprinter (−5%; Fig. 4*E*), 13% greater than subelite sprinters, and 21% greater than long-term resistance-trained individuals.

**Figure 5.**
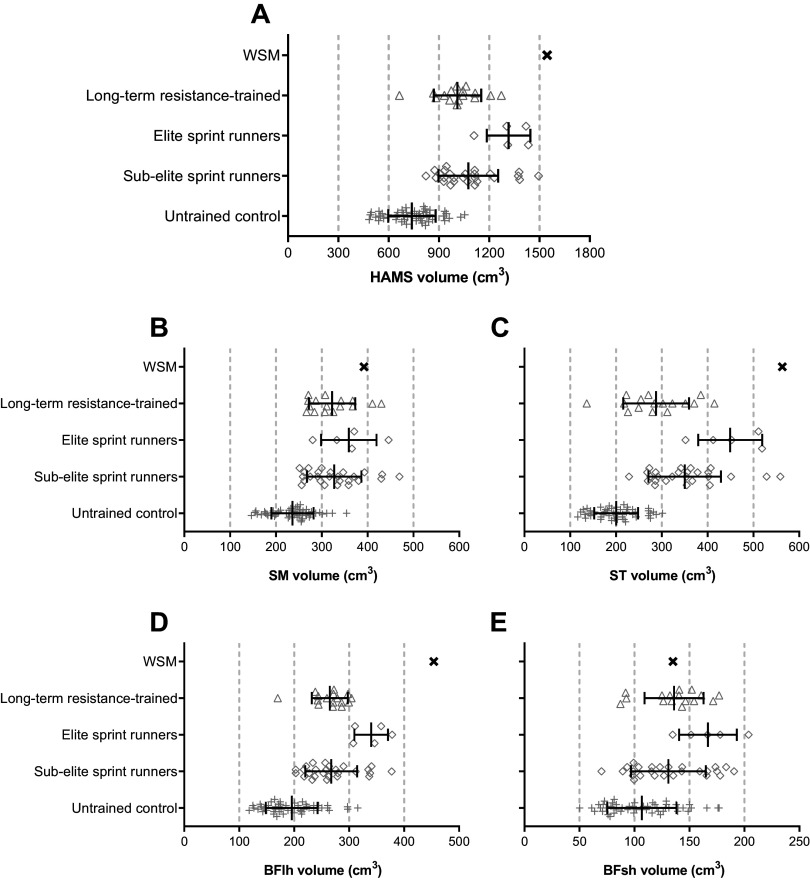
Overall hamstrings (HAMS; *A*), semimembranosus (SM; *B*), semitendinosus (ST; *C*), biceps femoris long head (BFlh; *D*), and biceps femoris short head (BFsh; *E*) muscle volume of a World's Strongest Man and deadlift champion (WSM) compared with long-term resistance trained [*n* = 16, from the work by Maden-Wilkinson et al. (10)], elite sprint runners [*n* = 5, from the work by Miller et al. (13)], subelite sprint runners [*n* = 26, from the work by Miller et al. (13)], and untrained control populations [*n* = 50, pooled population from the works by Miller et al. (13) (*n* = 11) and Balshaw et al. (14) (pretest data *n* = 39)].

Overall hamstring volume of the WSM (1,545 cm^3^) was 109% greater than a large pooled population of untrained controls (739 ± 142 cm^3^; *n* = 50), 44% greater than subelite sprinters (1,075 ± 178 cm^3^), 53% greater than long-term resistance-trained individuals (1,011 ± 142 cm^3^), and 17% greater than elite sprinters (1,315 ± 130 cm^3^; Fig. 5*A*). The WSM’s hamstring volume was also marginally larger (+3%) than the most muscular individual we have previously assessed (subelite sprinter, 1,495 cm^3^). The ST (563 cm^3^) and BFlh (454 cm^3^) volumes of the WSM were 132–182% larger than that of the pooled population of untrained controls (ST: 200 ± 48 cm^3^; BFlh: 196 ± 47 cm^3^; Fig. 5, *C* and *D*) and greater than the mean of any trained/athletic group we have previously assessed (Fig. 5, *C* and *D*). SM (392 cm^3^) volume of the WSM was 66% greater than untrained controls (SM 236 ± 46 cm^3^) and greater than the mean for trained/athletic groups we have previously assessed (Fig. 5*B*). BFsh volume (135 cm^3^) of the WSM was a modest 26% greater than that of our pool of untrained control participants (107 ± 31 cm^3^; Fig. 5*E*) but smaller than that of both long-term resistance-trained individuals (−1%; 136 ± 27 cm^3^) and elite sprinters (−19%; 167 ± 26 cm^3^; Fig. 5*E*).

### Patella Tendon Cross-Sectional Area and Moment Arm

**Figure 6.**
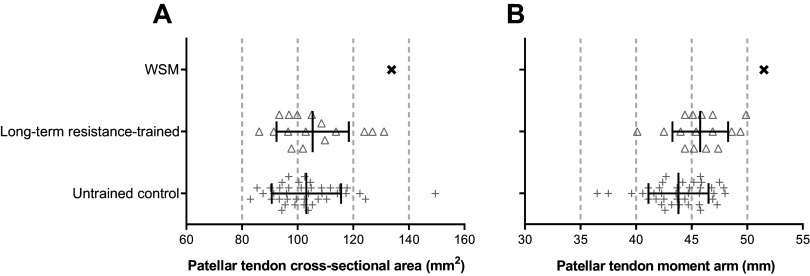
Patellar tendon mean cross-sectional area (*A*) and patellar tendon moment arm (*B*) of a World's Strongest Man and deadlift champion (WSM) compared with long-term resistance trained [*n* = 16, from the work by Massey et al. (15)] and untrained control populations [*n* = 39, from the work by Massey et al. (15)].

The patellar tendon mean CSA of the WSM (133.8 mm^2^) was larger than that of average untrained (+30%; 103.2 ± 12.5 mm^2^) and long-term resistance-trained individuals (+27%; 105.4 ± 13.0 mm^2^; Fig. 6*A*) but was smaller than the largest individual we have measured from these groups (149.5 mm^2^). The WSM’s patellar tendon moment arm (51.5 mm) was also larger than that of average untrained (+18%; 43.8 ± 2.7 mm) or long-term resistance-trained groups (+12%; 45.8 ± 2.5 mm; Fig. 6*B*) as well as being 3% greater than the highest individual moment arm we have previously assessed within these groups (49.9 mm).

## DISCUSSION

This study is the first to document the lower-body muscle and tendon morphology of a World's Strongest Man and deadlift champion (i.e., an exceptionally strong individual), and these are presented alongside functional whole body assessments, which exceeded the highest IMTP force (gross and net) and CMJ power values previously reported by 54%, 100%, and 164%, respectively. The WSM had overall lower-body muscularity approximately twice that of untrained controls (+96%) and 32% greater than that of elite 100-m sprinters. However, there was substantial anatomical variability in the magnitude of the differences, ranging from the plantar flexors (+120% vs. untrained) to the hip flexors (+65% vs. untrained). Similarly, some specific muscles, such as the guy rope muscles that stabilize the femur and pelvis, were 2.5–3.0 times the volume of untrained individuals (gracilis +140%, semitendinosus +157%, and sartorius +202%) but others displayed more marginal differences (BFsh +23%, iliopsoas +32% vs. untrained). Considering the knee extensors, the WSM had both quadriceps femoris volume greater than or equal to twofold that of untrained controls and a greater patella tendon moment arm than we have previously measured (+18% vs. untrained), which would be expected to combine to facilitate extraordinary strength. Furthermore, despite the WSM’s extremely large quadriceps femoris, their patellar tendon CSA was only 30% greater than that of untrained controls and not outside the range of tendons we have previously assessed. The results of this study provide novel insights into the muscle and tendon characteristics, as well as the strength and power capabilities, of an extraordinarily strong individual that may be toward the upper limit of human variation in these characteristics.

Although it was anticipated that the WSM would possess a larger total lower-body muscle volume/mass than untrained controls and other athletic/trained groups we have previously measured, the magnitude and pattern of the differences were unknown. The results indicated that the total volume of the measured muscles was almost twice that of average untrained participants and 32–63% larger than subelite and elite sprinters. Pronounced development of the antigravity muscles (i.e., hip extensors, knee extensors, and plantar flexors) was perhaps not that surprising given the WSM’s background in heavy lifting events (including being a double deadlift world champion and record holder). However, the hip flexors appear less important in these tasks, possibly explaining their more modest size, which was inferior to that of three elite 100-m sprinters we have previously assessed. The WSM’s plantar flexors were particularly large relative to untrained controls (+120%). This could be due to the plantar flexors being the smallest of the antigravity muscle groups that may experience very high mechanical stress and, thus, a pronounced adaptive stimulus during heavy lifting, carrying, and pulling tasks. Furthermore, the very heavy and, therefore, low-velocity nature of these tasks may limit the contribution of the stretch-shortening cycle and tendon recoil to the positive/concentric work done by the plantar flexors, potentially placing a higher demand on the contractile apparatus than for running and jumping tasks.

Considering individual muscles/compartments, the muscular development of the WSM was distinctly nonuniform. It is striking that the largest muscles relative to the untrained control population were the three “guy ropes” (sartorius, gracilis, and semitendinosus: +140–202%). These three muscles provide stability to the pelvis and femur by having origins at diverse points around the pelvis while sharing a common insertion onto the anteromedial tibia [via pes anserinus, the conjoined tendons of these three muscles (39)]. Large guy rope muscles likely enhance stabilization of the femur and pelvis and would be expected to be critical during heavy weight-bearing tasks. In contrast, the WSM’s five smallest muscles (relative to untrained controls) consisted of two hip flexors (iliopsoas and RF) and two monoarticular knee flexors; actions that appear far less important for lifting, carrying, and pulling tasks.

The WSM’s quadriceps volume and patellar tendon moment arm were both greater than that of untrained controls and indeed any individual we have previously measured. However, the magnitude of difference, relative to the untrained controls, was noticeably larger for quadriceps femoris volume (greater than or equal to twice as large) than for patellar tendon moment arm (+18%). Therefore, of these two key strength determinants, muscle size, rather than joint leverage, appeared to be the predominant factor responsible for the WSM’s extraordinary strength. Indeed, when we previously compared the muscle morphology and joint mechanics of individuals with distinct maximum strength capacity (long-term resistance-trained individuals vs. untrained controls), muscle size was the primary factor separating the groups with much more subtle differences in moment arm (10). The extreme example of muscle size provided by the WSM’s quadriceps femoris also gave the opportunity to investigate the scaling of tendon size to muscle size; extreme muscular size (greater than or equal to twice that for untrained controls) might be expected to be accompanied by comparable tendinous tissue size to effectively transmit high muscular forces to the skeleton. However, the WSM’s patellar tendon CSA was only 30% larger than untrained controls and within the range of individuals we have previously measured (Fig. 6*A*). This observation supports the notion that tendon structure may be largely fixed by adulthood (40), with only slow/limited changes in response to functional overload/resistance training. For example, we previously found patellar tendon CSA to show very subtle changes after 15 wk (45 training sessions) of heavy resistance training [+1.4% (41)] and no differences between long-term resistance-trained individuals and untrained controls (15).

### Limitations

Although the current investigation provides a detailed assessment of an individual at/toward the upper limit of human strength performance, it is important to appreciate study limitations. First, the participant was not measured immediately before their World's Strongest Man championship success or other landmark performances, and it is entirely possible the functional and structural characteristics we assessed may have been even higher directly prior to peak performances. Despite using a wide-bore MRI scanner, due to the size of the WSM’s shoulders and arms, it was not possible to scan their upper body. Thus, we were not able to investigate this aspect of the WSM’s muscle morphology; although given that greater hypertrophy occurs in the upper body compared with the lower body (42), it is possible that the WSM’s upper-body muscle size relative to untrained controls may have been even more pronounced than what we have documented for the lower body. In the current study to provide the most representative data on untrained control participants, the largest available untrained control populations were used for each category of measurements. Thus, different untrained control populations were used [e.g., comparison of quadricep and hamstring size (*n* = 102) vs. comparison of all the leg muscles (*n* = 11)], which led to some subtle discrepancies in the contrasts between these groups and the WSM [e.g., quadriceps femoris/knee extensors, +127% and +99% relative to our large pooled (*n* = 102) and smaller (*n* = 11) untrained control samples, respectively]. Importantly, however, this discrepancy does not appear to meaningfully affect the interpretation of the findings. There were subtle differences in the precise scanning and analysis approaches used with the reference populations featured in this study, including *1*) magnetic field strength [1.5 T (10, 11, 15) vs. 3.0 T, WSM and (13, 14)]; *2*) the interslice distance used to quantify quadriceps femoris and hamstrings muscle volume [1.5 cm (10, 11, 14) vs. 2.0 cm, WSM and (13)]; *3*) the calculation of muscle volume [area under the cubic spline ACSA-muscle length curve: (10, 11, 14) vs. the equation detailed earlier: WSM and (13)]; and *4*) the use of unilateral MRI measures derived from one limb (10, 11, 14, 15) or collapsed across two limbs [WSM and (13)]. However, it seems likely that these subtle differences would have had at most a very minor effect on the findings. Finally, it is also important to highlight that the differences documented between the WSM and comparative populations for the various measures included in the current study cannot be assumed to be anything other than a combination of both innate (genetic) and environmental (training and nutrition) factors.

### Conclusions

In conclusion, this novel investigation documented the muscle and tendon morphology and whole body strength and power characteristics of an exceptionally strong individual, relative to comparative athletic, trained, and untrained populations. Overall leg muscle volume of the WSM was approximately twice that of untrained controls but with pronounced anatomical variability in the extent of muscular development. The plantar flexor muscle group and the guy rope muscles (sartorius, gracilis, and semitendinosus: +140 to +202%), which stabilize the pelvis and femur, demonstrated the largest differences. The pronounced quadriceps femoris size of the WSM (greater than or equal to twice that of untrained) was accompanied by a more modest difference in patella tendon moment arm (+18%) and was not matched by a proportional difference in tendon size (+30%).

## DATA AVAILABILITY

Data will be made available upon reasonable request.

## SUPPLEMENTAL MATERIAL

10.6084/m9.figshare.26152939 Supplemental Material: https://doi.org/10.6084/m9.figshare.26152939.

## DISCLOSURES

No conflicts of interest, financial or otherwise, are declared by the authors.

## AUTHOR CONTRIBUTIONS

T.G.B. and J.P.F. conceived and designed research; T.G.B., G.J.M., R.M., E.J.M., and J.P.F. performed experiments; T.G.B., G.J.M., R.M., E.J.M., and T.M.M.-W. analyzed data; T.G.B. and J.P.F. interpreted results of experiments; T.G.B. prepared figures; T.G.B. and J.P.F. drafted manuscript; T.G.B. and J.P.F. edited and revised manuscript; T.G.B., G.J.M., R.M., E.J.M., T.M.M.-W., and J.P.F. approved final version of manuscript.
